# Deficiency of BAP1 inhibits neuroblastoma tumorigenesis through destabilization of MYCN

**DOI:** 10.1038/s41419-023-06030-5

**Published:** 2023-08-05

**Authors:** Xiaoling Zhang, Xianling Cong, Xiangting Jin, Yu’e Liu, Tong Zhang, Xinyuan Fan, Xiyao Shi, Xiaoying Zhang, Xue Wang, Yong-Guang Yang, Xiangpeng Dai

**Affiliations:** 1 Key Laboratory of Organ Regeneration and Transplantation of Ministry of Education, First Hospital, Jilin University, Changchun, China; 2 National-Local Joint Engineering Laboratory of Animal Models for Human Disease, First Hospital, Jilin University, Changchun, China; 3 Department of Dermatology, China-Japan Union Hospital of Jilin University, Changchun, China; 4 Tongji University Cancer Center, Shanghai Tenth People’s Hospital of Tongji University, School of Medicine, Tongji University, Shanghai, China; 5 International Center of Future Science, Jilin University, Changchun, China

**Keywords:** Cancer epigenetics, Cancer epigenetics, Oncogenes

## Abstract

The transcription factor MYCN is frequently amplified and overexpressed in a variety of cancers including high-risk neuroblastoma (NB) and promotes tumor cell proliferation, survival, and migration. Therefore, MYCN is being pursued as an attractive therapeutic target for selective inhibition of its upstream regulators because MYCN is considered a “undruggable” target. Thus, it is important to explore the upstream regulators for the transcription and post-translational modification of MYCN. Here, we report that BRCA1-associated protein-1 (BAP1) promotes deubiquitination and subsequent stabilization of MYCN by directly binding to MYCN protein. Furthermore, *BAP1* knockdown inhibits NB tumor cells growth and migration in vitro and in vivo, which can be rescued partially by ectopic expression of MYCN. Importantly, depletion of *BAP1* confers cellular resistance to bromodomain and extraterminal (BET) protein inhibitor JQ1 and Aurora A kinase inhibitor Alisertib. Furthermore, IHC results of NB tissue array confirmed the positive correlation between BAP1 and MYCN protein. Altogether, our work not only uncovers an oncogenic function of BAP1 by stabilizing MYCN, but also reveals a critical mechanism for the post-translational regulation of MYCN in NB. Our findings further indicate that BAP1 could be a potential therapeutic target for MYCN-amplified neuroblastoma.

## Introduction

Neuroblastoma (NB), the most common pediatric extracranial solid tumor, is reported to arise from the undifferentiated neural crest cells [1, 2]. NB accounts for about 13% mortality of all pediatric cancer patients [1, 3]. In detail, the 5-year survival rate for children with low-risk and intermediate-risk NB is higher than 95% and around 90% to 95%, respectively. However, children with high-risk NB only have a 5-year survival rate of about 50% [4]. The amplification and active mutation of various oncogenes that drive the development of NB are frequently found in high-risk NB patients. Among them, MYCN, a member of the MYC oncogene family, is highly associated with high-risk NB, poor prognosis, and overall short survival time of NB patients [5]. Furthermore, the frequency of *MYCN* amplification in neuroblastoma patients is around 20–30% and currently, amplification of *MYCN* is thought to be the best genetic characteristic to stratify high-risk NB [5]. Importantly, depletion of *MYCN* in *MYCN*-amplification NB induces the tumor cell apoptosis, differentiation and cell growth retardation, indicating that inhibiting MYCN by targeting its transcription and protein stability might be a therapeutic strategy for *MYCN*-amplification NB [6, 7]. However, MYCN is considered a “undruggable” target, because MYCN proteins, like MYC, have two extended alpha-helices and do not provide suitable surfaces for small molecular to bind which limited the development of strategies to directly targeting MYCN proteins [5]. Currently, various strategies developed to block MYCN include: (1) inhibiting *MYCN* expression by blocking *MYCN* transcription regulators (e.g., epigenetic reader proteins, such as BET protein BRD4), (2) targeting upstream regulators regulating MYCN protein stability, (3) promoting NB cell apoptosis via activating p53 pathway, and (4) inducing cell differentiation [5]. For example, the BET protein inhibitor JQ1 could retard the growth of *MYCN*-amplified NB in the patient-derived xenografts (PDX) models and the TH-*mycn* transgenic mouse model. The NB cell-bearing mice treated with JQ1 demonstrated an extended overall survival time, and JQ1 treatment also induced tumors apoptosis, retarded cells proliferation as well as decreased the expression of *MYCN* [8]. Given that Aurora A Kinase has the capability to stabilize MYCN in a kinase activity-independent manner and small-molecule inhibitors could efficiently bind to its ligand binding sites, blocking Aurora A Kinase by inhibitors could be a potential strategy for NB patients with *MYCN*-amplified tumors [9, 10]. However, drug resistances to current chemotherapy treatments are frequently observed in most high-risk NB patients who initially showed response to chemotherapy [11]. Importantly, it was reported that the levels of *MYCN* expression could affect the sensitivity to chemotherapy [12]. Consistently, our previous study indicated that the targeted protein BRD4 abundance confers resistance to BET inhibitor JQ1 [13]. Therefore, more new insights into the mechanisms of regulating MYCN expression or protein stability will not only help to drive development of new therapeutic strategies to combat *MYCN*-amplified NB, but also help to understand the underlying mechanisms for drug resistance during the treatment of NB patients which will benefit the development of strategies for personalized medicine.

Protein ubiquitination, an important type of post-translational modification (PTM), plays a crucial role in regulating multiple cellular processes by controlling protein stability, trafficking and protein-protein interaction [14]. Furthermore, protein ubiquitination is reversibly controlled by the E3 ubiquitin ligases and deubiquitinases (DUBs) through ubiquitinating and deubiquitinating substrates respectively. Therefore, it is not surprising that by deubiquitinating and stabilizing various key oncoproteins, DUBs play crucial roles in the development and progress of cancers. BRCA1-associated protein-1 (BAP1), a ubiquitin C-terminal hydrolase (UCH), was firstly identified as a DUB to mediate BRCA1 stability [15]. Recently, accumulating evidence have revealed that BAP1 mutations are frequently found in different cancer types which indicated that BAP1 might be a critical tumor suppressor. However, more recent published studies indicated that the tumor suppressor function of BAP1 is controversial and might be cell type- and context-dependent [16–21]. Germline mutations of BAP1 demonstrated a better prognosis in some cancer types but somatic mutations caused a worse prognosis in other cancer types [22]. Moreover, BAP1 was found to stabilize the transcription factor KLF5 and subsequently promoted the breast cancer cell proliferation and tumor growth in vitro and in vivo [23]. Therefore, it is an urgent need to explore more specific substrates of BAP1 which will help to validate the fundamental biology functions of BAP1 in cancers including neuroblastoma and to accurately develop novel therapeutic strategies for cancers patients with abnormal expression or mutation of BAP1.

Here, we characterized a molecular mechanism and translational significance of BAP1-mediated deubiquitination of MYCN in neuroblastoma. Inhibiting BAP1 by shRNA-mediated knockdown decreases the expression of MYCN largely through reducing the BAP1-mediated deubiquitination of MYCN. We further validated the crucial role of BAP1 in tumor promotion by regulating MYCN. Moreover, depletion of BAP1 confers cellular resistance to JQ1 and Alisertib. These results together demonstrated a pivotal role of BAP1 in its oncogenic function in NB and uncovered a novel regulator for MYCN which is highly correlated with drugs resistance in *MYCN* -amplified NB cells.

## Results

### BAP1 is a DUB to maintain MYCN stability by deubiquitinating MYCN

**Fig. 1:**
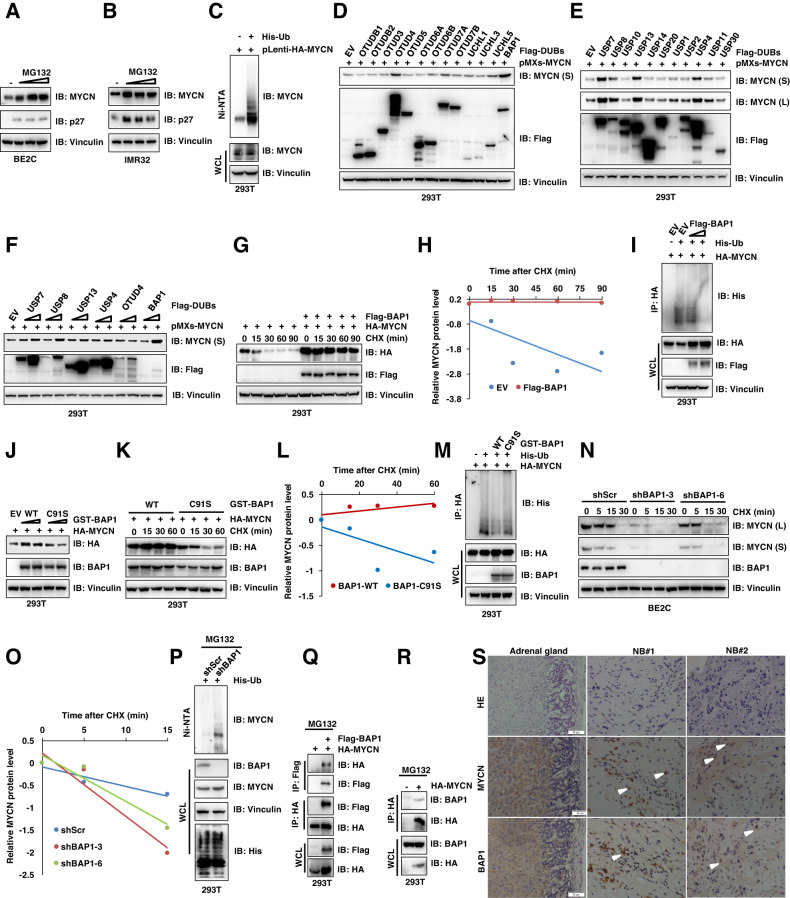
BAP1 deubiquitinates and controls MYCN protein stability. **A**, **B** Immunoblot (IB) analyses of whole-cell lysate (WCL) from BE2C (**A**) and IMR32 (**B**) cells treated with different concentration of MG132 for 10 h. **C** In vivo ubiquitination analysis of WCL and His pulldown of 293T cells transfected with the indicated plasmids. Forty hours after transfection, cells were treated with 20 μM MG132 for 6 h before harvesting. Ni-NTA, nickel-nitrilotriacetic acid. **D**–**F** IB analysis of WCL derived from 293T cells transfected with the indicated plasmids. **G** IB analysis of WCL derived from 293T cells transfected with HA-MYCN and Flag-BAP1 constructs. Cells were treated with 100 μg/ml cycloheximide (CHX) and collected at the indicated time points. **H** MYCN protein abundance in (**G**) was quantified by ImageJ and plotted as indicated (**H**). **I** Immunoblot analyses of WCL and immunoprecipitates from 293T cells transfected with plasmids expressing the indicated proteins. Twenty-four hours after transfection, cells were treated with MG132 (10 μM) for 10 h before they were harvested. His-Ub histidine-tagged ubiquitin. **J** IB analysis of WCL derived from 293T cells transfected with HA-MYCN and GST-BAP1-WT/C91S plasmids. **K** IB analysis of WCL derived from 293T cells transfected with HA-MYCN and GST-BAP1-WT/C91S constructs. Cells were treated with 100 μg/ml cycloheximide (CHX) and collected at the indicated time points. **L** MYCN protein abundance in (**K**) was quantified by ImageJ and plotted as indicated (**L**). **M** Immunoblot analyses of WCL and immunoprecipitates from 293T cells transfected with plasmids expressing the indicated proteins. Twenty-four hours after transfection, cells were treated with MG132 (10 μM) for 10 h before they were harvested. His-Ub, histidine-tagged ubiquitin. **N** *BAP1*-knockdown cells (shBAP1–3 and shBAP1-6) as well as control BE2C cells (shScr) were treated with 100 μg/ml cycloheximide (CHX) for the indicated time period before they were harvested. Equal amounts of WCL were immunoblotted with the indicated antibodies. **O** MYCN protein abundance in (**N**) was quantified by ImageJ and plotted as indicated (**O**). **P** In vivo ubiquitination analysis of WCL and His pulldown of 293T cells transfected with the His-Ub plasmids. Forty hours after transfection, cells were treated with 20 μM MG132 for 6 h before harvesting. Ni-NTA, nickel-nitrilotriacetic acid. **Q**, **R** Immunoblot (IB) analyses of whole-cell lysate (WCL) and immunoprecipitates (IP) derived from 293T cells transfected with indicated constructs. Thirty hours post-transfection, cells were treated with 10 μM MG132 for 10 h before harvesting. **S** Representative images for immunohistochemical staining of BAP1 and MYCN in human NB tumors and adrenal gland. Scale bars, 50 μm.

Dysregulation of MYCN in cancers lead to aberrant MYCN oncoprotein levels and will cause dramatic transcriptional changes of its downstream genes, which subsequently promote the tumorigenesis. Therefore, block of MYCN is thought to be a therapeutic approach in MYCN-driven cancers but the development of pharmacological inhibitors directly targeting MYCN proteins has been challenging. MYCN belongs to the “undruggable” target due to its unfriend protein structure for the binding of small molecules. Therefore, targeting the upstream regulators of MYCN have been considered therapeutic concepts to specifically target MYCN-driven tumors [24]. It is crucial to understand how MYCN protein stability is regulated and whether MYCN protein abundance affects cellular resistance to therapeutic inhibitors. To this end, we observed that, in SK-N-BE-2-C (BE2C) and IMR32 neuroblastoma cell lines, treatment with the 26 S proteasome inhibitor MG132 led to a marked increase in endogenous MYCN protein abundance (Fig. 1A, B). Mechanistically, we found that MYCN could be subjected to polyubiquitination in cells (Fig. 1C). These findings indicated that the ubiquitin-mediated pathways are involved in controlling MYCN protein stability (Fig. 1A, B), which is consistent with the results published previously [2, 25].

Importantly, by deubiquitinating and stabilizing some key oncoproteins, DUBs play crucial roles in the development of various cancers. Given that small-molecule inhibitors have been currently developed to target the enzymatic activity of DUBs, and some of them are moving forward into preclinical studies or clinical trials [14]. We further want to explore whether DUBs are involved in regulating the MYCN protein stability and the cellular sensitivity of the drugs targeting MYCN. First, we screened a panel of DUBs belong to ovarian tumor (OTU) superfamily and the ubiquitin C-terminal hydrolase (UCH) superfamily by co-transfecting them into 293T cells with MYCN. We found that the OTUD4 and BAP1, but not other DUBs we examined, dramatically elevated MYCN protein abundance (Fig. 1D). Moreover, BAP1, but not OTUD4, upregulated the MYCN protein level in a dose-dependent manner (Supplementary Fig. 1A). Second, we further screened the ubiquitin-specific protease (USP/UBP) superfamily DUBs to identify whether other kinds of DUBs are involved in regulating MYCN protein levels [2]. Interestingly, we found the USP4, USP7, USP8 and USP13, but not other DUBs we examined, dramatically elevated MYCN protein abundance (Fig. 1E). However, further experiment indicated that only BAP1 and USP7 could elevate the MYCN protein level in a dose-dependent manner, but BAP1 have the best effect on the elevation of MYCN protein levels (Fig. 1F). Therefore, given the better effect of BAP1 in elevating MYCN protein levels in our experimental settings, the BAP1 will be our major focus in the remainder of the study.

Next, we performed the protein half-life assay and ubiquitination assay to further confirm whether BAP1 is a bona fide DUB for MYCN to plays a vital role in positively regulating MYCN protein abundance. Cycloheximide (CHX) was used to inhibit protein translation and the MYCN protein half-life was measured (Fig. 1G, H). Ectopic expression of BAP1 dramatically extended the half-life of ectopically expressed MYCN (Fig. 1G, H). To directly check whether MYCN protein stability is regulated by BAP1-dependent deubiquitination, the Flag-BAP1, His-Ub and HA-MYCN were co-transfected into 293T cells. MG132 was used to treat the cells for 10 h before harvesting for in vivo ubiquitination. Notably, BAP1 could reduce the polyubiquitination of MYCN (Fig. 1I), which might be the underlying mechanism for the effect of BAP1 on extend half-life of MYCN. Furthermore, we expressed wild-type (WT) BAP1 and catalytic inactive mutant of BAP1 (C91S) [26] in 293T cells (Fig. 1J). The results showed that BAP1-WT, but not BAP1-C91S, dramatically elevated the MYCN protein levels (Fig. 1J). In line with this finding, the BAP1-C91S failed to extended the half-life of MYCN (Fig. 1K, L). Mechanistically, in vivo ubiquitination results indicated that BAP-WT, but not BAP1-C91S, could markedly decreased the polyubiquitination of MYCN protein in 293T cells (Fig. 1M).

In keeping with the critical role of BAP1 in governing the MYCN protein stability in cells, shRNAs for BAP1 were utilized to genetically deplete endogenous BAP1 in BE2C cells and the cells were treated with CHX to inhibit protein translation to perform the half-life assay (Fig. 1N, O). The result showed that depletion of BAP1 dramatically reduced the BAP1 protein levels and decreased the half-life of endogenous MYCN (Fig. 1N, O), suggesting that MYCN is a putative BAP1 downstream deubiquitination substrate, as least in the experimental setting we examined. Furthermore, His-Ub was transfected into the 293T cells in which endogenous *BAP1* was depleted by shBAP1 and the in vivo ubiquitination was performed on the cells after being treated by MG132 for 6 h (Fig. 1P). The polyubiquitination of endogenous MYCN proteins were detected using an anti-MYCN antibody and depletion of BAP1 markedly increased the MYCN protein polyubiquitination (Fig. 1P).

In support of the notion that BAP1 might be an upstream DUB for MYCN, Flag-BAP1 and HA-MYCN were co-transfected into 293T cells and the immunoprecipitation (IP) assay was performed to examine whether BAP1 will interact with MYCN. The IP result showed BAP1 specifically interacts with MYCN (Fig. 1Q). Furthermore, endogenous BAP1 could specifically interacts with MYCN in 293T cells (Fig. 1R). In line with this finding, immunofluorescence staining results confirmed that both MYCN and BAP1 are co-localized in the nucleus in 293T cells (Supplementary Fig. 1B). The ubiquitin E3 ligases and DUBs together controlled the reversible process of ubiquitination/deubiquitination. It was reported that E3 ligase SCF^Fbw7^ could negatively regulate MYCN protein stability through the ubiquitination of MYCN [27]. Therefore, next we sought to examine whether BAP1 has the capability to protect MYCN from FBXW7-mediated degradation as a DUB of MYCN (Supplementary Fig. 1C, D). The results indicated that FBXW7 could efficiently promote the degradation of MYCN, but BAP1 could antagonize the degradation effect of FBXW7 and markedly rescued the protein levels of MYCN in a dose-dependent manner (Supplementary Fig. 1C, D). The immunohistochemistry (IHC) staining results further confirmed that BAP1 correlated with MYCN expression in NB patient samples and their expression was upregulated in NB patient samples compared with the embryonic adrenal tissue (Fig. 1S). Taken together, these data suggest that the BAP1 is likely an upstream DUB of MYCN to deubiquitinate and stabilize MYCN protein.

### Depletion of *BAP1* inhibits neuroblastoma cell growth

**Fig. 2:**
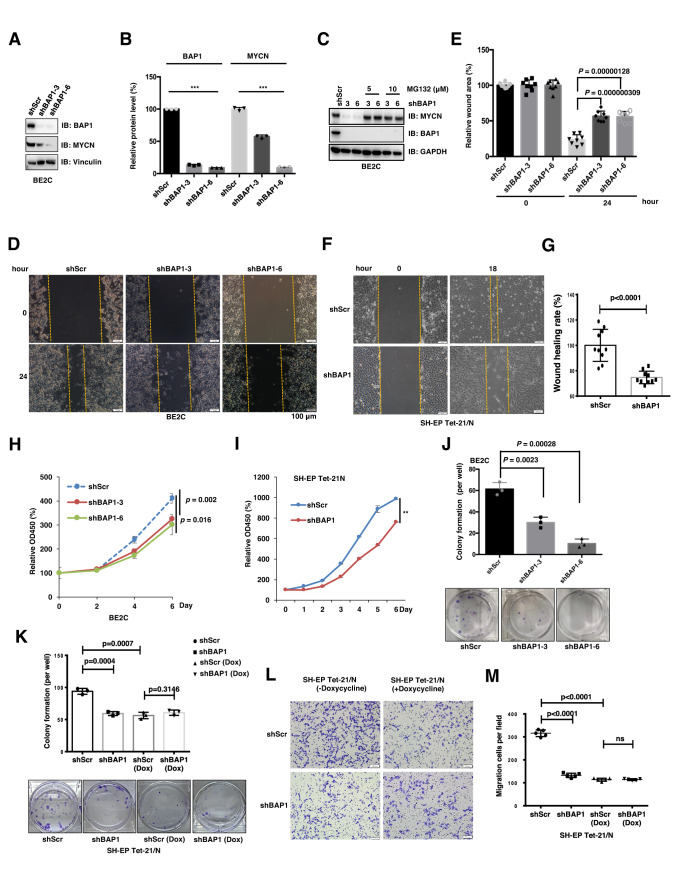
BAP1 deficiency inhibits NB cell growth in vitro. **A** Immunoblot (IB) analyses of whole-cell lysate (WCL) derived from the BE2C cells infected with indicated lentiviral sgRNAs. The infected cells were selected with 2–3 μg/ml puromycin for 72 h to eliminate non-infected cells before they were harvested. **B** Quantification of the MYCN and BAP1 protein intensities in (**A**). **C** IB analysis of WCL derived from BE2C cells infected with the indicated BAP1 shRNAs. Cells were treated with or without indicated concentration of MG132 for overnight before they were harvested. **D** Representative images for the wound-healing assays of shBAP1 BE2C cells. The wound edges are indicated by yellow lines. Scale bar, 100 μm. **E** The quantitative results of (**D**) (*n* = 8). The *y* axis represents the relative wound area. Error bars represent s.d. from eight repeats. *t* test. **F** Representative images for the wound-healing assays of shBAP1 SH-EP Tet21/N cells (MYCN amplification). The wound edges are indicated by yellow lines. Scale bar, 100 μm. **G** The quantitative results of (**F**) (*n* = 10). The *y* axis represents the relative wound area. Error bars represent s.d. from ten repeats. *t* test. **H**, **I** Growth curves of BE2C (**H**) cells and SH-EP Tet21/N cells (**I**) with shRNA-mediated *BAP1* knockdown (shBAP1). **P < 0.01. **J** Colony-formation assay. Depletion of endogenous *BAP1* in BE2C cells displays moderate decrease in colony-formation ability. The number of colonies were counted and quantified. Data are shown as mean ± SD for three independent experiments. *P* value was indicated in figure, *t* test. BE2C cells were infected with indicated lentiviral shRNAs. The infected cells were selected with 2 μg/ml puromycin for 72 h to eliminate non-infected cells before they were used for colony-formation assay. **K** Colony-formation assay. Depletion of endogenous *BAP1* in SH-EP Tet21/N cells decreases the colony-formation ability. However, upon depletion of MYCN by the Dox treatment, the colony-formation ability of SH-EP Tet21/N+Dox cells is not changed after depletion of endogenous *BAP1*. The number of colonies were counted and quantified. Data are shown as mean ± s.d. for three independent experiments. *P* value was indicated in the figure, *t* test. SH-EP Tet21/N cells were infected with indicated lentiviral shBAP1. The infected cells were selected with 2 μg/ml puromycin for 72 h to eliminate non-infected cells before they were used for colony-formation assay. **L** Representative images (*n* = 5) of migrated SH-EP Tet21/N cells infected with the indicated lentiviral shBAP1. The SH-EP Tet21/N cells were treated with or without Dox before plated for the transwell migration assay. Scale bar, 100 μm. **M** Quantification of the migrated cells in (**L**). In all plots, data are shown as mean ± s.d. for five independent experiments.

MYCN plays an important role in NB development by activating the transcription of various genes responsible for proliferation, survival, metastasis, self-renewal, and angiogenesis and inhibiting the genes involved in cell cycle arrest, immune surveillance, differentiation, antagonizing metastasis and angiogenesis. Among these functions, promotion of proliferation and cell cycle progression is the best characterized tumorigenic effect of MYCN in NB [5]. However, the function of BAP1 in regulating tumorigenesis is controversial and appears to be tissue or cellular context-dependent. Importantly, the physiological role of BAP1 in neuroblastoma remains largely unexamined which urges us to examine the roles of BAP1 in NB. Endogenous *BAP1* was efficiently knocked down by two short hairpin RNAs (shBAP1–3/−6) which was confirmed by WB (Fig. 2A, B). Consistently, *BAP1* knockdown markedly induced MYCN protein reduction in BE2C cells (Fig. 2A, B). Interestingly, the decrease of MYCN protein upon *BAP1* knockdown in BE2C cells could rescued by treatment of MG132, which further confirmed the BAP1 regulates MYCN protein levels as a DUB by modulating the proteasome progress and ubiquitination status of MYCN (Fig. 2C and Supplementary Fig. 2A).

Next, the function of BAP1 on migration, proliferation and colony formation of BE2C cells was further examined (Fig. 2D–M). The results of wound-healing assays indicated that compared with the shScr control cells, depletion of *BAP1* in BE2C and SH-EP Tet21/N cells dramatically decreased the cells migration (Fig. 2D–G). Subsequently, depletion of *BAP1* in BE2C and SH-EP Tet21/N cell lines led to a significant decrease in cell proliferation (Fig. 2H, I) and in cell transformation ability as evidenced by a reduction in the number of colonies formed in tissue culture dishes (Fig. 2J, K). Moreover, the transwell migration and invasion assay results indicated that depletion of *BAP1* in SH-EP Tet21/N dramatically decrease the migration ability of NB cells (Fig. 2M). Interestingly, the colony formation, migration and cell proliferation assays results indicated that *BAP1* knockdown inhibited non-inhibitory effect on the SH-EP Tet21/N cells with doxycycline induced *MYCN* depletion (Fig. 2K–M and Supplementary Fig. 2B–D). Taken together, BAP1 knockdown exhibits a dramatic anti-oncogenic function in *MYCN*-amplified NB cells by inhibiting their migration, proliferation and colony-formation ability.

### Depletion of *BAP1* inhibits NB cells growth in vitro and in vivo in part through destabilizing MYCN

**Fig. 3:**
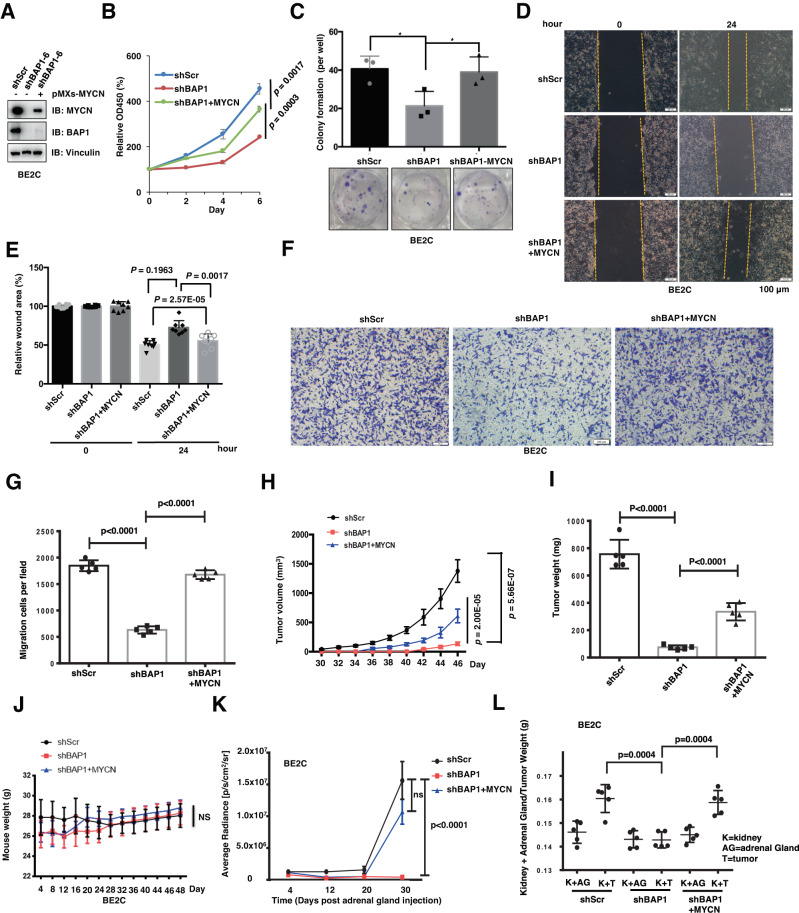
Deficiency of BAP1 inhibits NB cell growth in vitro and in vivo through MYCN. **A** Immunoblot (IB) analyses of whole-cell lysate (WCL) from shBAP1 BE2C cells stably expressing MYCN. **B** Growth curves of BE2C cells with shRNA-mediated *BAP1* knockdown (shBAP1) and stably expressing MYCN. **C** Colony-formation assay. Depletion of endogenous *BAP1* in BE2C cells displays moderate decrease in colony-formation ability and overexpression of MYCN will partly recover the colony-formation ability. The number of colonies were counted and quantified. Data are shown as mean ± SD for three independent experiments. *P* value was indicated in figure, *t* test. BE2C cells were infected with indicated lentiviral shRNAs and retroviral MYCN. **D** Representative images for the wound-healing assays of BE2C cells with BAP1 knockdown and MYCN overexpression. The wound edges are indicated by yellow lines. Scale bar, 100 μm. **E** The quantitative results of (**D**) (*n* = 8). The *y* axis represents the relative wound area. Error bars represent s.d. from eight repeats. *t* test. **F** Representative images (*n* = 5) of migrated BE2C cells infected with the indicated lentiviral shBAP1 and/or stable overexpression of MYCN. Scale bar, 100 μm. **G** Quantification of the migrated cells in (**F**). In all plots, data are shown as mean ± s.d. for five independent experiments. **H** In vivo tumor growth was monitored at the indicated time points. **I** The weights of the dissected tumors in Supplementary Fig. 3C. **J** The body weights of the tumor-bearing mice were measured at the indicated time points. **K** In vivo orthotopic tumor growth was monitored over the indicated time period by detecting and analyzing the bioluminescence signals. **L** The weight of kidney + adrenal gland and kidney + tumor in Supplementary Fig. 3E (*n* = 5).

Given that BAP1 could maintain the MYCN protein stability and depletion of *BAP1* could hamper the BE2C and SH-EP Tet21/N cells growth, we intended to further investigate whether the oncogenic function of BAP1 in NB cells is mediated by MYCN. Therefore, we stably overexpressed MYCN in *BAP1*-depleted BE2C cells (Fig. 3A). Notably, ectopic expression of MYCN in *BAP1*-depleted BE2C cells could significantly promote their proliferation (Fig. 3B) as well as colony formation (Fig. 3C), in part due to that the protein abundance of MYCN could be efficiently elevated upon ectopic expression of MYCN (Fig. 3A). Consistently, MYCN overexpression in *BAP1*-depleted BE2C cells dramatically increased cell migration in the wound-healing assays (Fig. 3D, E) and transwell migration and invasion assays (Fig. 3F, G).

Furthermore, we investigated the inhibitory effect of BAP1 knockdown in BE2C cells in vivo utilizing the subcutaneous xenograft mice models implanted with shScr, shBAP1, and shBAP1+MYCN BE2C cells (Fig. 3H–J and Supplementary Fig. 3A–C). The bioluminescence intensity was measured to monitor the tumor growth in early stage before their volumes are large enough to observe (Supplementary Fig. 3A, B). Consistent with in vitro cellular results, depletion of *BAP1* significantly retarded the growth of tumor xenografts, whereas overexpression of MYCN partially rescued *BAP1* depletion-mediated cells growth inhibition (Supplementary Fig. 3B). Furthermore, tumor volume and weight were measured and analyzed in the later stage at the indicated time points (Fig. 3H, I). The tumor growth patterns in later stage are consistent with that in early stage and depletion of *BAP1* in BE2C cells markedly retarded tumor growth and overexpression of MYCN in shBAP1 cells dramatically increased the tumor growth (Fig. 3H, I). Furthermore, the body weight of subcutaneously xenografted mice are not significantly affected by the implanted BE2C cells (Fig. 3J). Importantly, to furthermore precisely examine the inhibitory effect of *BAP1* knockdown on NB cells growth, we constructed the orthotopic xenograft mice model by implanting the BE2C cells into one of the adrenal glands of mice (red arrow, Supplementary Fig. S3E). The bioluminescence intensity was measured to monitor the tumor growth in the indicated time points (Fig. 3K and Supplementary Fig. 3D). Notably, consistent with in vitro cellular results and subcutaneous xenograft results, depletion of *BAP1* significantly inhibits the growth of implanted tumor cells, but overexpression of MYCN partially rescued *BAP1* depletion-mediated cells growth retardation (Fig. 3K and Supplementary Fig. 3D). Furthermore, the weight of tumor plus kidney exhibited a consistent result with subcutaneous xenograft that *BAP1* depletion markedly reduced the weight but overexpression of MYCN partially rescued *BAP1* depletion-caused weight reduction (Fig. 3L). Moreover, the fact that *BAP1* knockdown could not affect *MYCN* non-amplification SH-EP Tet21/N+Dox cells colony formation and migration strengthens our notion that BAP1 exerts its oncogenic function in NB cells in a MYCN-dependent manner (Fig. 2K–M). These results collectively demonstrated a physiological function for MYCN in mediating the inhibitory effect of *BAP1* knockdown on hampering the NB cell proliferation and tumorigenesis in vitro and in vivo.

To further explore the oncogenic function of BAP1 in NB, we performed a serial of in vitro cellular and in vivo tumor xenograft assays using the BAP1 overexpressed BE2C cells (Supplementary Fig. S4). However, although the MYCN-mediated inhibitory effect of *BAP1* deficiency on the growth of *MYCN*-amplified NB cells was significant and solid which is confirmed by biochemistry assays (Fig. 1), in vitro cellular biological assays (Fig. 2) and in vivo mice xenograft assays (Fig. 3), the results generated from the BAP1 overexpression BE2C cells are unappreciated. We stably overexpressed *BAP1* in BE2C cells (Supplementary Fig. 4A) which exhibits a mild upregulation of MYCN protein level which is consistent with the result of BAP1 knockdown. Overexpression of BAP1 cause a growth inhibition at late culture stage and decrease colony formation (Supplementary Fig. 4B, C). In in vivo orthotopic xenograft assays showed a reduced weight of kidney plus tumor (Supplementary Fig. 4D–F) without the significant body weigh change for the tumor-bearing mice (Supplementary Fig. 4G). Moreover, similar results with that done by BE2C cells were generated from the cell proliferation assays, colony-formation assays and migration assays (Supplementary Fig. 4I–L) using the BAP1 overexpressed SH-EP Tet21/N cells which also displayed a moderate upregulation of MYCN protein level upon BAP1 overexpression (Supplementary Fig. 4H). The slight upregulation of MYCN protein level in BPA1 overexpressed NB cells and other substrates of BAP1 which exert predominant function in the BAP1 overexpression setting might be the underlying mechanisms for BAP1 expression-induced cell growth retardation. For instance, BAP1 overexpression could induce apoptosis to retard BE2C cells proliferation by releasing Bax through 14–3–3 protein interaction and reducing the Bcl-2 expression regardless of BAP1 DUB activity [28]. However, the detailed mechanisms are extremely complicated which deserves further in deep investigation by *TH*-BAP1 conditional transgenic or knockout NB mice models.

### *BAP1* deficiency confers cellular resistance to BET inhibitor JQ1 and Aurora A kinase inhibitor Alisertib

**Fig. 4:**
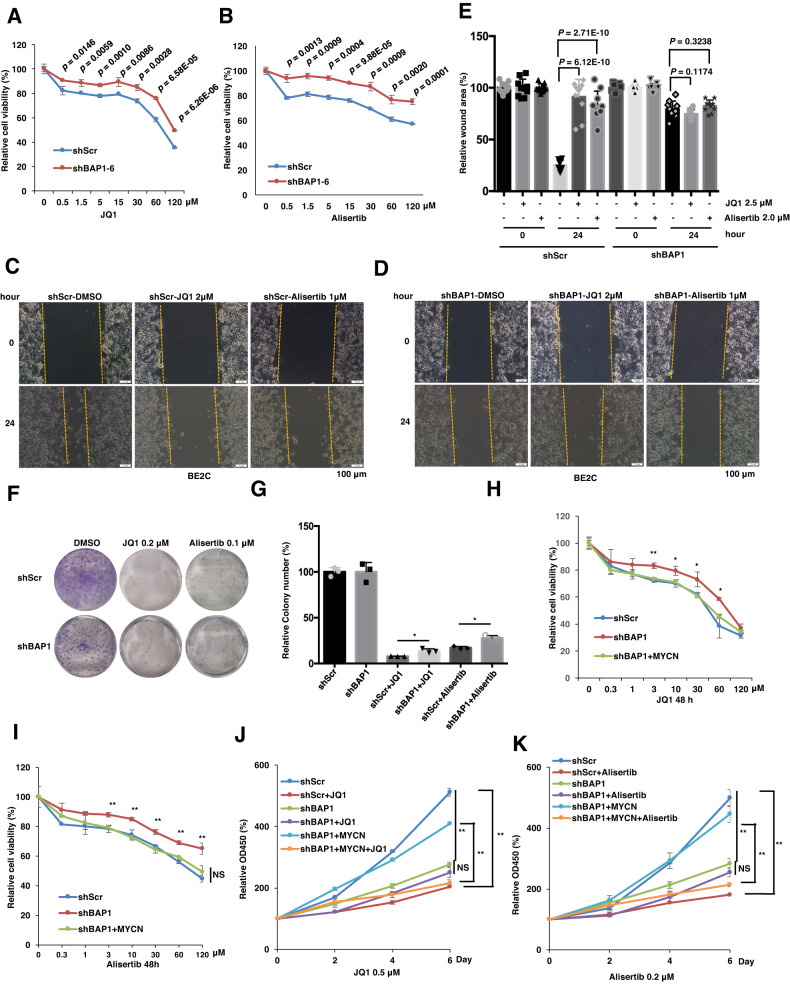
*BAP1* knockdown conferred cellular resistance to BET inhibitor JQ1 and Aurora kinase inhibitor Alisertib in NB. **A**, **B** Cell viability of BE2C cells with shRNA-mediated *BAP1* knockdown (shBAP1) that were treated with indicated concentration of JQ1 (**A**) and Alisertib (**B**) for 24 h. shScr BE2C cells were used as control. *P* values are shown in figure, *t* test. **C**, **D** Representative images for the wound-healing assays of *BAP1*-knockdown (shBAP1) BE2C cells with or without JQ1 (**C**) and Alisertib (**D**) treatment. The wound edges are indicated by yellow lines. Scale bar, 100 μm. **E** The quantitative results of (**C**, **D**) (*n* = 8). The *y* axis represents the relative wound area. Error bars represent SD from eight repeats. *t* test. **F**, **G** Colony-formation assay. knockdown of endogenous *BAP1* in BE2C cells displays moderate resistance to JQ1 and Alisertib treatment in colony-formation ability. The number of colonies were counted and quantified. The colony number of shScr and shBAP1 without drug treatment groups are set up to “100%”, then the colony number of the drug treatment groups were compared with the relative colony number of shScr and/or shBAP1. Data are shown as mean ± SD for three independent experiments. *P < 0.05, *t* test. BE2C cells were infected with indicated lentiviral shRNAs. The infected cells were selected with 2 μg/ml puromycin for 72 h to eliminate non-infected cells before they were used for colony-formation assay. **H**, **I** Cell viability of BE2C cells with shRNA-mediated *BAP1* knockdown (shBAP1) and stably expressing MYCN that were treated with the indicated concentration of JQ1 (**H**) and Alisertib (**I**) for 48 h. shScr BE2C cells were used as control. *P < 0.05, **P < 0.01, *t* test. **J**, **K** Growth curves of BE2C cells with shRNA-mediated *BAP1* knockdown (shBAP1) and stably expressing MYCN that were treated with or without 0.5 μM JQ1 (**J**) and 0.2 μM Alisertib (**K**) for 6 days. shScr BE2C cells were used as control. **P < 0.01, *t* test.

Given that MYCN, one of the MYC-family members, is basic helix-loop-helix (bHLH) transcription factor without catalytic domain, direct targeting MYCN is limited and approaches to inhibit MYCN activity are developed by indirectly targeting proteins regulating MYCN protein stability or expression [12]. Currently, targeting MYCN through inhibiting bromodomain-containing proteins and Aurora Kinase A in various cancers is well studied [8, 10, 29, 30]. However, the frequently emerged cancer drug resistance limited their therapeutic effect on cancer patients and the underlying mechanisms for drug resistance are multifaceted. To this end, it was reported that the triple-negative breast cancer (TNBC) cell lines with higher expression level of *MYCN* are more sensitive to BET inhibitor [12] and in prostate cancer cells, elevated BRD4 protein level confers resistance to JQ1 [13]. Therefore, it is critical to examine whether dysregulation of MYCN protein stability caused by BAP1 depletion or mutation may affect the sensitivity of NB cells to Aurora Kinase A or BET inhibitors. Interestingly, we found that the BE2C cells depletion of endogenous *BAP1* by shRNA-mediated knockdown were relatively less sensitive to the BET inhibitor JQ1 (Fig. 4A) and Aurora A kinase inhibitor Alisertib (Fig. 4B). In line with this finding, depletion of endogenous *BAP1* inhibited JQ1- and Alisertib-induced suppression of BE2C cells migration (Fig. 4C–E) and colony formation (Fig. 4F, G). Furthermore, the results of cell viability assay indicated that additional overexpression of MYCN re-sensitized *BAP1*-depleted BE2C cells to JQ1 and Alisertib treatment (Fig. 4H–I). Moreover, Furthermore, the results of cell proliferation assay showed that ectopic expression of MYCN in shBAP1 cells could restore the sensitivity of *BAP1*-depleted BE2C cells to JQ1 and Alisertib treatment (Fig. 4J, K). Altogether, these results suggested that BAP1-depleted BE2C cells acquired resistance to BET inhibitors and Aurora Kinase A inhibitor largely through the decreased MYCN protein abundance. Hence, our results suggest that the decreased protein levels of MYCN caused by depletion of *BAP1* might contribute the resistance to BET inhibitors and Aurora Kinase A inhibitors in NB cells.

### BAP1 is positively correlated with MYCN in neuroblastoma

**Fig. 5:**
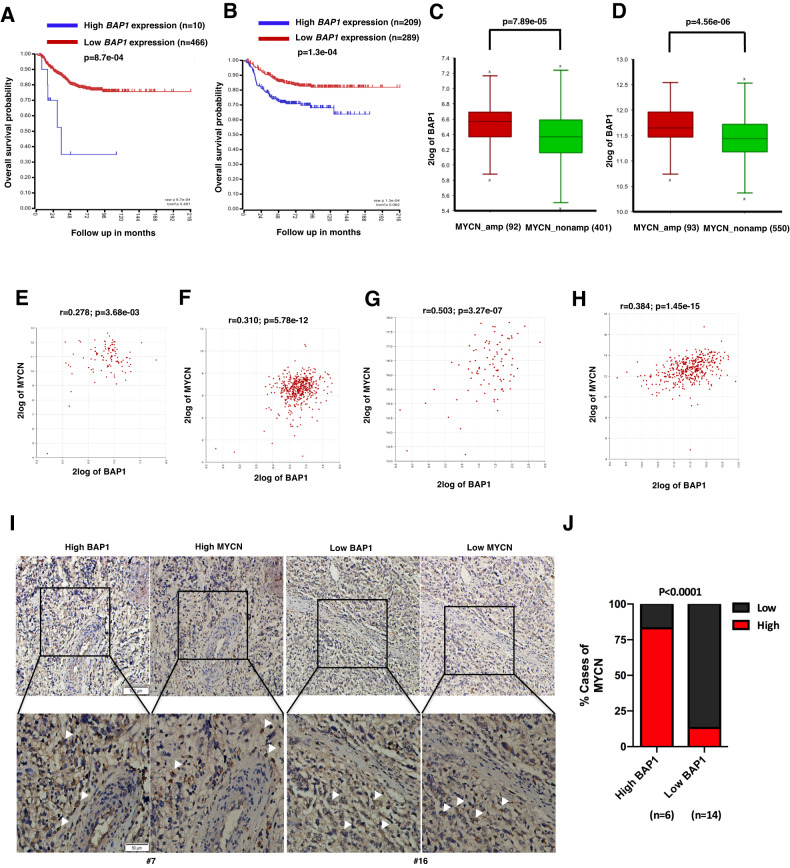
The clinical relevance of BAP1 and MYCN mRNA expression in human neuroblastoma. **A**, **B** Kaplan–Meier survival curves showed a significant association between NB patient survival and BAP1 mRNA expression in the two human NB databases. **A** GEO: GSE45547 dataset, 649 neuroblastoma tumors were generated using 44 K oligonucleotide microarrays. **B** GEO: GSE62564 dataset, RNA-seq data, expressed in reads per million (RPM) for each gene, for 498 clinically annotated primary neuroblastoma samples (SEQC neuroblastoma cohort). **C**, **D** BAP1 expression between MYCN-amplified (MA) and MYCN non-amplified (MNA) patients. Adjusted *P* value is from one-way analysis of variance (ANOVA), followed by the Benjamini–Hochberg method. **C** GSE45547 dataset, *n* = 649, MA vs. MNA = 93 vs. 550, *P* = 4.56e-06. **D** GSE62564 dataset, *n* = 498, MA vs. MNA = 92 vs. 401, *P* = 7.89e-05. **E**–**H** Correlation of BAP1 and MYCN mRNA levels in neuroblastomas. Two datasets were analyzed including the gencode19 cohort for neuroblastomas–Westermann (*n* = 579) (**E**, **F**) and GEO: GSE49710 (*n* = 498) (**G**, **H**). Correlation of BAP1 and MYCN mRNA levels in high-risk NB with MYCN amplification, p = 0.05 (**E**), p = 3.27e-07 (**G**), and in NB with MYCN non-amplification, *P* = 5.78e-12 (**F**), *P* = 1.45-15 (**H**). **I** Representative images for immunohistochemistry staining of BAP1 and MYCN in human NB tumors array. Scale bars, 10× (upper),100 μm; 20× (lower), 50 μm. **J** Positive correlation between BAP1 and MYCN protein levels in samples from NB tissue array. The tissue array consists of 22 NB samples which were classified into two groups (high BAP1, 6; low BAP1 14) according to the BAP1 protein level. *P* < 0.0001, chi-square test.

Last, the correlation between the expression of *BAP1* and *MYCN*, the expression of *BAP1* and overall survival in NB patients were further analyzed. The Kaplan–Meier survival curves indicated a significant correlation between NB patient survival and *BAP1* mRNA expression in the two human NB databases (Fig. 5A, B). NB Patients with relatively high levels of *BAP1* demonstrated poorer overall survival than those NB patients with low levels of *BAP1* (Fig. 5A, B). Furthermore, *BAP1* expression between *MYCN*-amplified (MA) and *MYCN* non-amplified (MNA) patients was analyzed from two human NB databases and the results showed that the *MYCN*-amplified NB patients showed a higher *BAP1* expression level than that in non-amplified (MNA) NB patients (Fig. 5C, D). Importantly, results from the larger-scale array of two datasets (*n* = 579 or 498) showed that *BAP1* and *MYCN* mRNA levels were significantly correlated in high-risk NB with *MYCN* amplification (Fig. 5E, G) and in NB with *MYCN* non-amplification (Fig. 5F, H). To further evaluate the clinical relevance and correlation of BAP1 and MYCN, we performed BAP1 and MYCN immunohistochemistry staining on NB tissue microarray and observed a markedly positive correlation between BAP1 and MYCN levels (Fig. 5I, J). Therefore, these results suggested that the expression of BAP1 and MYCN are dramatically correlated in NB patients.

## Discussion

**Fig. 6:**
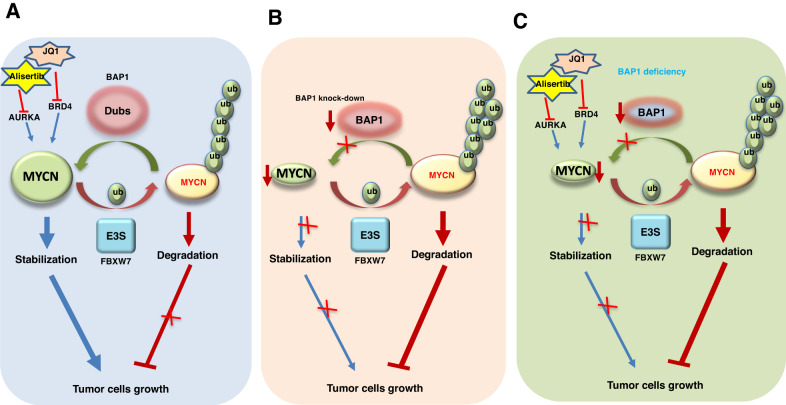
The proposed model of BAP1 promotes MYCN stabilization and mediates the drug sensitivity of NB cells. **A** BAP1 promotes MYCN deubiquitination and stabilization as a deubiquitinase of MYCN. The NB cells with wide-type BAP1 are sensitive to JQ1 and Alisertib. **B** Downregulation of BAP1 by shRNA or siRNA to reduce MYCN level will retard the NB cell proliferation and growth. **C** Deficiency of *BAP1* in NB cells confers resistance to JQ1 and Alisertib in a MYCN-dependent manner.

In this study, we identified BAP1 as a bona fide deubiquitylase (DUB) for MYCN to govern MYCN protein stability. Same as other post-translational modifications, ubiquitination/deubiquitination is also a reversible biological event which is controlled by ubiquitin E3 ligases and DUBs. E3 ubiquitin ligases such as FBXW7 could promote MYCN ubiquitination and degradation, however, BAP1 deubiquitinates MYCN and increases its stability. Dysregulation of BAP1 leads to a decreased MYCN protein level and subsequently causes NB cell growth inhibition (Fig. 6) and resistance to JQ1 and Alisertib treatment (Fig. 6).

As MYCN amplification is now regarded as a biomarker to feature the high-risk NB [5], MYCN is thought to be an important therapeutic target in *MYCN*-amplification NB [6, 7]. However, the characteristic of MYCN protein structure make MYCN as a “undruggable” target for small molecular and it is challenged to develop strategies directly targeting MYCN protein [5]. Therefore, various strategies were developed to inhibit MYCN by targeting upstream regulators regulating MYCN transcription and protein stability [5]. Given the critical role of MYCN in the development and progress of high-risk neuroblastoma, there is no doubt that MYCN could be controlled by multiples regulators at the transcriptional and post-translational levels. The DUBs USP3 and USP7 were reported to stabilize MYCN by promoting its deubiquitination [2, 31]. FBXW7 and HUWE1 are the two E3 ubiquitin ligases of MYCN and promote MYCN ubiquitination and degradation [25, 32]. Aurora A Kinase could disturb the binding between FBXW7 and MYCN to stabilize MYCN [10]. Furthermore, PLK1-induced auto-ubiquitination of FBXW7 inhibits FBXW7-mediated proteasomal degradation of MYCN [33]. Here, we demonstrate that BAP1 could bind and deubiquitinate MYCN to increase the stability of MYCN. Consistently, depletion of *BAP1* in MYCN-amplified BE2C and SH-EP Tet21/N cells but not MYCN non-amplified SH-EP Tet21/N+Dox cells, inhibited cell proliferation and migration in vitro (Fig. 2) and retarded tumor growth in subcutaneous and orthotopic xenograft mice models (Fig. 3 and Supplementary Fig. 3) in a MYCN-dependent manner.

It was well known that ubiquityation and related processes play important role in the physiology and pathology of human cells and defects in the modulation of ubiquitylation lead to a variety of diseases. Therefore, dysregulation of DUBs contributes to various diseases including cancers. BAP1 is mostly considered a transcriptional co-activator, but BAP1 can also exhibit the transcriptional co-repression function. Therefore, deficiency of *BAP1* will upregulate or downregulate a bundle of genes correlated with apoptosis, DNA repair, cell metabolism, cell cycle, and cell survival [34]. Notably, the mutation or deletion of *BAP1* are frequently found in melanoma, renal cell carcinoma and mesothelioma which implied a tumor suppressor activity of BAP1 [35]. But the exact function of BAP1 as intrinsic oncogene or tumor suppressor is not fully understood [36]. Germline mutations of BAP1 exhibit a favorable prognosis in some cancer types but somatic mutations are related with worse prognosis in other cancer types [22]. Moreover, BAP1 is a DUB for the transcription factor KLF5 and depletion of *BAP1* inhibits breast cancer cell proliferation and tumor growth in vitro and in vivo in an KLF5-dependent manner [23]. Therefore, the exact physiological and pathological role of BAP1 depend on cell or tissue context and cancer type which might be affected by the cell microenvironment. It is worthwhile to note that, some tumor suppressors including LKB, TSC1, and TSC2 also exhibit tumor cell promotion function especially in some metabolic stresses conditions [37, 38]. Hence, it is not surprising that BAP1 exhibited a prosurvival role and attenuated the metabolic stress-induced cell death by repressing the transcription of the activating transcription factor 3 (ATF3) and C/EBP homologous protein (CHOP) in a DUB activity-dependent manner [39]. Furthermore, *BAP1* knockout in clear cell renal cell carcinoma (ccRCC) inhibits cell growth and migration and promotes the mesenchymal-epithelial transition (MET) partially by downregulating Snail and reducing Rho family GTPases activity [40]. In line with this notion, we found BAP1 knockdown could inhibit *MYCN*-amplified NB cells proliferation, migration and tumor growth in BE2C and SH-EP Tet21/N cells and in xenograft mice models (Figs. 2 and 3) which support the notion that BAP1 might be an oncogene in NB. Importantly, unlike BAP1 has a high percentage of mutation in other cancer types, we did not find any mutation reports in NB in the database of cBioPortal.com. Since MYCN is a certified genetic marker for high-risk NB and its expression is related to poor survival of NB patients, we sought to check whether BAP1 could be a potential biomarker for NB patients (Fig. 5). Interestingly, high expression level of *BAP1* is significantly associated with poor overall survival of NB patients with amplified *MYCN* (Fig. 5). Therefore, BAP1 might be serve as a potential target for high-risk NB with amplified *MYCN*.

However, Sime et al. found that BAP1 overexpression could also induce apoptosis to retard BE2C cells proliferation by releasing Bax through 14–3–3 protein interaction and reducing the Bcl-2 expression regardless of BAP1 DUB activity [28]. But *BAP1* deficiency cause apoptosis in primary keratinocytes, mouse embryonic stem cells, or E1A oncogene-immortalized mouse embryonic fibroblasts by downregulating the BAP1 target genes Bcl-2 and Mcl1 [41]. To further explore the detailed effect of BAP1, we constructed BE2C cells and SH-EP Tet21/N cells stably overexpressed BAP1 and these cells showed moderate elevated MYCN protein abundance (Supplementary Fig. S4) which phenocopied our *BAP1* knockdown results that the MYCN protein abundance is dramatically reduced upon *BAP1* knockdown (Figs. 1 and 2). The results further confirmed that BAP1 might be the bona fade deubiquitylase of MYCN. Although the MYCN protein was elevated upon BAP1 overexpression, our results of in vitro cells assay and in vivo xenograft assay using the BAP1 overexpressed BE2C and/or SH-EP Tet21/N cells demonstrated that, BAP1 overexpression could also inhibit cell proliferation, migration, colony formation and tumor growth which is similar with the results of BAP1 knockdown. Although it was well known that the role of BAP1 as a tumor suppressor or oncogene is cellular and tissue context or cancer-type dependent, this is the first time to find that knockdown and overexpression of same gene in one tumor type have same phenotype. These results implied that the underlying mechanisms for the role of BAP1 in regulating cell behavior are extremely complicated. Also, the results indicated that the gene profiles of gene knockdown might not be consistent with the same gene overexpression which means the downregulated genes upon gene knockdown might not be the same genes which are upregulated upon gene overexpression. Furthermore, it might be that MYCN is the predominant gene mediated the inhibitory effect of *BAP1* knockdown, but BAP1 overexpression have little promoting effect on the MYCN-amplified NB cells due to the very high level of MYCN in the cells. Therefore, the predominant gene mediated the inhibitory effect of BAP1 overexpression might be the Bcl-2, BAX or other apoptosis related genes. It was reported that depletion of BAP1 in cells with normal expression level of BAP1 could decrease the cell growth and lead to a delayed G1 to S1 transition [34]. It was also found that in trophically stressed neuroblastoma cells, overexpression of MYCN could weaken the anti-apoptotic effect of Bcl-2 and increase the cell death [42].

Moreover, the difference of experiment methods and *BAP1*-knockdown stretagy between us and Sime et al. might be the reason for the contradictory result of *BAP1* knockdown. Sime et al. found that SK-D-NZ cells with low expression of endogeous of BAP1 grow faster than the SK-N-F1 cells with high expression of BAP1 which did not consider the genetic diversiy of different cell lines. Furhermore, they use the BAP1 siRNA to cause *BAP1* knockdown in SK-N-F1 cells which used for the cell apoptosis assay but not used the cell proliferation, migration and xenograft assay [28]. Compared with their BAP1 knockdown assays, we have tons of results indicated that shRNA mediated knockdown inhibit BE2C and SH-EP Tet21/N cells proliferation, migration, invasion, colony formation and tumor growth in the subcutaneous and orthotopic xenograft mice models.

It is well studied that the BET protein inhibitor JQ1 and Aurora A Kinase inhibitor Alisertib could efficiently inhibit cell growth of *MYCN*-amplified tumors [8–10]. However, most high-risk patients will develop resistance to various chemotherapy treatments [43]. Consistently, NB cells could be resistant to JQ1 through elevating the expression of PI3K signal [44]. We previously demonstrated that in prostate cancer, higher BRD4 proteins levels could confer resistance to JQ1 [13]. Moreover, the *MYCN* expression status could affect the sensitivity of triple-negative breast cancer (TNBC) cells to JQ1 and the cells with lower MYCN expression were less sensitive to JQ1 [12]. Therefore, the underlying mechanisms for drug resistance is complicated and might be drug-type or cancer-type dependent. Consistent with the results reported in TNBC [12], we found that depletion of *BAP1* in BE2C cells confer resistance to JQ1 and Alisertib in a MYCN-dependent manner (Fig. 4). The possible reason might be the cells with lower level of MYCN are less responsive to the treatment of JQ1 and Alisertib which lost their targets. Importantly, recent developed BAP1 inhibitor iBAP-II could significantly reduce the small cell lung cancer (SCLC) cell viability and suppress tumor growth in vivo which proved an evidence for the oncogenic function of BAP1 and blocking BAP1 activity could be a novel therapeutic strategy [45]. Furthermore, they also found that iBAP-II significantly downregulated the genes involved in MYC-family targets which partly supports our notion that MYCN stability is regulated by BAP1 (Fig. 6). However, it was found that the small cell lung cancer (SCLC) cells with depleted BAP1 was resistant to iBAP-II [45]. Therefore, targeting BAP1 by its inhibitors such as iBAP-II or in combination with other therapeutic strategies might be an effective treatment strategy for human neuroblastomas with amplified *MYCN* which warrants further in deep investigation.

Hence, our findings not only reveal a molecular mechanism for the oncogenic function of BAP1 and the regulation of MYCN stability but also provide molecular rationale for the clinical exploration of novel strategies to combat NB in *MYCN*-amplified patients by targeting the deubiquitylase BAP1 (Fig. 6).

## Materials and methods

### Cell culture, transfections, and infections

HEK293T, HEK293FT, SK-N-BE-2-C (BE2C), SH-EP Tet21/N cell (Kind gift of Dr. Frank Westermann, German Cancer Research Center) and IMR32 cells were cultured in DMEM medium supplemented with 10% FBS, 100 units of penicillin and 100 μg/ml streptomycin. The doxycycline (Dox) in the culture medium is used to regulate the *MYCN* expression of SH-EP Tet21/N cells. *MYCN* is not expressed (MYCN off) upon the addition of doxycycline while MYCN is expressed (MYCN on) without doxycycline in the medium [46]. Lipofectamine 2000 (Invitrogen) were used for the cell transfection according to the instruction. Cycloheximide (CHX) assays were performed as described previously [47].

### Plasmids and shRNAs

pLenti-HA-MYCN was subcloned the MYCN cDNA into pLenti-HA-hygro vector. pLenti-BAP1 was subcloned the BAP1 cDNA into pLenti-hygro vector. GST-BAP1-WT, GST-BAP1-C91S, pBabe-BAP1-WT, shBAP1–3 and shBAP1-6 constructs were kind gifts from Dr. Ceshi Chen (Kunming Institute of Zoology, Kunming, Yunnan). shScr and His-Ub were provided by Dr. Wenyi Wei (Beth Israel Deaconess Medical Center, Harvard Medical School, Boston, MA). pMXs-MYCN was purchased from Addgene. Flag-Myc-OTUB1/OTUB2, Flag-OTUD3/D4/D5/D6A/D6B/D7A/D7B, Flag-UCHL1/UCHL3/UCHL5, Flag-BAP1, Flag-USP1/2/4/7/8/10//11/13/14/20/30 and His-Ub constructs have been described previously [48]. Details of plasmid constructions are provided upon request.

### Antibodies

All the primary antibodies were used at a 1:1000 dilution, and secondary antibodies were diluted at 1:3000 dilution in 5% nonfat milk for immunoblotting assay. Anti-BAP1 (sc-28383), anti-N-Myc (sc-53993) and Polyclonal anti-HA (Y-11) were purchased from Santa Cruz Biotechnology. Anti-His (2365 S) antibodies were purchased from Cell Signaling. Monoclonal anti-HA antibody (MMS-101P) was purchased from Covance. Polyclonal anti-Flag antibody (F-2425), monoclonal anti-Flag antibody (F-3165, clone M2), anti-Tubulin antibody (T-5168), anti-Vinculin antibody (V-4505), anti-Flag agarose beads (A-2220), anti-HA agarose beads (A-2095), peroxidase-conjugated anti-mouse secondary antibody (A-4416) and peroxidase-conjugated anti-rabbit secondary antibody (A-4914) were purchased from Sigma.

### Immunohistochemistry

Immunohistochemistry (IHC) studies were performed on normal embryonic adrenal gland, neuroblastoma specimens and NB tissue array (Cat# N264001, Bioaitech Co., Ltd. Xi’an, China). The First Hospital of Jilin University obtained the informed consent from the patients for their NB tissues. All the patients whose NB tissues were collected and used in our experiment understood the scientific usage of their NB tissues. We did not use the tissue from the patients who did not sign the informed consent. The use of tissue samples was approved by the First Hospital of Jilin University. The specimens were fixed in 4% paraformaldehyde, embedded in paraffin and sliced into 10-μm-thick sections for further usage. The sections and tissue array were then subjected to the IHC staining using BAP1 (sc-28383) and MYCN (sc-53993) antibodies. The immunohistochemistry was performed as described previously [49] with slight modification according to the instruction of tissue array.

### Immunofluorescence assay

The 293T cells were transfected with Flag-BAP1 and HA-MYCN for 30 h and then were fixed in 4% paraformaldehyde for 30 min at room temperature. After three washes in PBS for 5 min each, the cells were permeabilized with 0.1% Triton X-100 in PBS and blocked with 5% bovine serum albumin, followed by incubation with the BAP1 and HA antibody for overnight. Cells were then rinsed for three times in PBS, and secondary antibodies conjugated with Alexa Fluor 488 and Alexa Fluor 568 (Invitrogen) were subsequently used, and the cells were incubated in the dark for 1 h at room temperature. After washing in PBS for three times, glass slides were covered by coverslips together with ProLong Gold antifade reagent added DAPI (4′,6-diamidino-2-phenylindole) (Invitrogen). All images were taken by LSM 880 confocal laser scanning microscope (Carl Zeiss, Oberkochen, Germany).

### In vivo His-pulldown ubiquitination assays

The assay was performed as described previously [13]. Briefly, His-ubiquitin and the indicated constructs were co-transfected into 293T cells. Thirty-six hours post-transfection, cells were treated with 20 μM MG132 for 5–6 h. Cells were then collected and lysed in buffer A (6 M guanidine-HCl, 0.1 M Na_2_HPO_4_/NaH_2_PO_4_, 10 mM imidazole, pH 8.0) and sonicated. Then the lysates were incubated with Ni-NTA matrices (Qiagen) for 3 h at room temperature. His pull-down products were then washed twice with buffer A, twice with buffer A/TI (1 volume buffer A and 3 volume buffer TI) and one time with buffer TI (25 mM Tris-HCl, 20 mM imidazole, pH 6.8). After washing in buffer TI, the pull-down proteins were resolved by SDS-PAGE for immunoblotting.

### In vivo ubiquitination assay

The assay was performed as described previously [13]. Briefly, HA-tagged ubiquitin and the desired constructs were co-transfected into 293T cells. Thirty-six hours post-transfection, cells were treated with 10 μM MG132 for overnight. Cells were collected and lysed in 200 μL denaturing buffer (1% SDS, 50 mM Tris pH 7.5, 0.5 mM EDTA and 1 mM dithiothreitol). After incubation at 100 °C for 10 min, the lysate was sonicated and diluted tenfold with EBC lysis buffer and incubated with anti-HA-conjugated agarose beads (Sigma, mouse antibody) for 4 h at 4 °C. Immunoprecipitants were washed five times with NETN buffer before they were resolved by SDS-PAGE and immunoblotted with the indicated antibodies.

### Immunoblots and immunoprecipitation

Cells were lysed in EBC lysis buffer (50 Mm Tris pH 8.0, 120 Mm NaCl, 0.5% NP-40) supplemented with protease inhibitors (Complete Mini, Roche) and phosphatase inhibitors (phosphatase inhibitor cocktail set I and II, Calbiochem). The protein concentrations of the supernatants of cell lysis were measured by TECAN SPARK spectrophotometer using the Pierce^TM^ BCA protein assay reagent (23227, ThermoFisher Scientific, USA). The lysates were then resolved by SDS-PAGE and immunoblotted with indicated antibodies. For immunoprecipitation assays, same amount of protein lysates was incubated with the appropriate antibody-conjugated beads (8 µL) for 4–5 h at 4 °C, or incubated with antibody (1–2 μg) overnight at 4 °C followed by the addition of Protein A sepharose beads (GE Healthcare) for an hour. Immunoprecipitants were then washed with NETN buffer (20 Mm Tris, pH 8.0, 100 Mm NaCl, 1 mM EDTA, and 0.5% NP-40) five times, then resolved by SDS-PAGE and immunoblotted with indicated antibodies. ImageJ software was used to quantify the immunoblot band intensity [13].

### Virus packaging and cell infection

The virus packaging and cell infection were performed described previously with some modifications [48]. For lentiviral shRNA infection, 293T cells were transfected with shScr or BAP1 shRNAs or pLenti-MYCN or pLenti-BAP1 vectors, together with packing vectors (∆8.9 and VSVG plasmids) using lipofectamine 2000 reagent according to the instruction. The cells were infected with the indicated virus and selected with medium containing puromycin and/or hygromycin for at least 72 h.

### Cell viability assays

The desired cell lines were seeded in 96-well plates (3 × 10^3^ cells/well) and cultured in 100 μL of DMEM medium containing 10% FBS. After 24 h, cells were treated with various concentrations of indicated compounds in 50 μL of medium for 24 to 48 h, and cell viability was measured using Enhanced Cell Counting Kit-8 (Beyotime Biotechnology) according to the manufacturer’s instructions. All cell viability experiments were conducted in triplicate.

### Cell proliferation assays

The indicated cells were seeded in 96-well plates (3 × 10^3^ cells/well) and cultured in 100 μL of DMEM medium containing 10% FBS. To test the effect of inhibitors, 24 h after seeding, cells were treated with various concentrations of inhibitors in 200 μL of DMEM medium for 6 days. At the indicated time points, cell proliferation was detected by Enhanced Cell Counting Kit-8 (Beyotime Biotechnology) according to the manufacturer’s instructions. Data are shown as mean ± SD from three independent experiments.

### Colony-formation assay

Cells were seeded in six-well plates (1 × 10^3^ cells/well) and cultured in DMEM medium with 10% FBS for one to 2 weeks, dependent on the size of the colony. To test the effect of inhibitors, 24 h after seeding, cells were treated with various concentrations of inhibitors in 3 mL of DMEM medium for one to two weeks. Then cells were fixed by fixing buffer (10% methanol and 10% acetic acid), and the colonies were stained with crystal violet and counted.

### Cell migration and invasion assays

Cells were seeded in six-well plates for wound-healing assays. The wound was created by 200-μL tip when cells are confluent. The wound area images were collected every 12 h under the microscope. The wound area in different time points are analyzed by ImageJ.

The transwell migration and invasion assays were performed as described previously with some modifications [13]. For transwell cell migration assays, 3 × 10^4^ to 1 × 10^5^ indicated NB cells in serum-free DMEM medium were plated on the top of the 8.0-μm 24-well plate chamber insert (Corning, 3422), and DMEM medium containing 10% FBS was added at the bottom of the insert. The assay for each sample was performed in triplicate. After 24 h’ culture, the cells were fixed with 4% paraformaldehyde for 15 to 30 min and washed with PBS for three times, a cotton swab was utilized to scrape the cells on the top of the insert. Then the 0.5% crystal violet blue was used to stain the cells adherent to the bottom of the insert for 15 to 30 min. Then the crystal violet blue was washed off with double-distilled water (ddH2O). The positive-staining cells were detected and recorded under the microscope. Data are shown as mean ± s.d. from three independent experiments.

### Animals

NOD-Prkdcem26Cd52Il2rgem26Cd22/Nju (NOD/SCID IL2rg−/− mice, NCG) mice (5–7 weeks) were purchased from Nanjing Biomedical Research Institute of Nanjing University. Mice were housed in SPF environment in the animal facility of First Hospital of Jilin University for one week before use. All care and treatment of experimental mice were performed under the guidelines outlined in the Guide for the Care and Use of Laboratory Animals. All procedures were approved by the Institutional Animal Care and Use Committee (IACUC) of the First Hospital of Jilin University.

### Mouse subcutaneous xenograft assays

In total, 1 × 10^6^ indicated BE2C cells were suspended in 100 μL of DMEM medium without FBS and injected into the flanks of NCG mice (six mice per group). Tumor growth was detected every 4 days by bioluminescence intensity (BLI) on the Caliper IVIS Lumina II in vivo imaging system from day 4 to day 24 after tumor implantation. Intraperitoneal injection of firefly D-fluorescein potassium salt (15 mg/kg) was given to the mice before in vivo imaging. The tumor growth curve was drawn based on the average bioluminescence intensity we detected. From 30 days after tumor cells implantation, tumor size was measured every 2 days with a caliper, and the tumor volume was calculated with the formula: *L* × *W*^*2*^ × 0.52, where L is the longest diameter and W is the shortest diameter of the tumor. At the end of the studies, mice were killed, and in vivo solid tumors were dissected and weighed.

### Mouse orthotopic xenograft assays

The mouse orthotopic xenograft assay using the indicated NB cells was performed as described previously [50]. The NCG mice (five mice per group) were anesthetized by the intraperitoneal injection of 0.05 g/mL tribromoethanol (35 mg/kg). Mice were fixed in the prone position on the surgical drape. The skin on the lower edge of the left rib was cut with ophthalmic scissors, and expand the wound to the desired size (~1 cm). Then an incision was created on the muscle above the spleen to expose the left kidney and adrenal gland. The indicated cells (1 × 10^5^ in 20 μL PBS in 1 mL insulin syringe) were carefully and slowly injected into the adrenal gland. The expanded fascia between the kidney and adrenal gland was the criteria for the successful injection. After surgery, gentamicin sulfate water (80 mg /250 mL drinking water) was supplied to the mice for a week to prevent possible infection. At the indicated time points, the tumor growth was measured by bioluminescence intensity (BLI) on the Caliper IVIS Lumina II in vivo imaging system. Each mouse received an intraperitoneal injection of D-fluorescein potassium salt (15 mg/kg) 7–8 min ahead of the in vivo imaging. The growth curve of the transplanted tumor was drawn according to the measured bioluminescence intensity.

### Quantitative RT-PCR

Total RNA was extracted using the RNeasy mini kit (Qiagen), and the reverse transcription reaction was performed using Power SYBR Green PCR Master Mix (ThermoFisher Scientific, 4367659). Real-time PCR was performed with the 7500 Fast Real-Time PCR system (ABI). Primers for MYCN were 5′-CCACAAGGCCCTCAGTACC-3′ and 5′-TCTTCCTCTTCATCATCTTCATCA-3′. Data are shown as mean ± SD for three independent experiments.

### Bioinformatics analyses

Microarray data for BAP1 and MYCN from 649 neuroblastoma samples (Kocak neuroblastoma cohort) were downloaded from R2 platform (http://r2.amc.nl). The matched clinical annotations containing overall survival data were utilized to construct Kaplan–Meier survival curves of subgroups dichotomized by median gene expression using the survival R package (v2.42.1). Survival data (death from disease, follow-up months) for each group (*BAP1*-low vs. *BAP1*-high expression) were extracted manually from GSE45547 (*n* = 649) and GSE62564 (*n* = 498) dataset and imported into the R Environment. A scanning procedure was performed (with a minimum group size of 8) and the cutoff value for most significant separation between groups with high or low expression of BAP1 was chosen to generate the curves. Kaplan–Meier analysis was performed using the survival package in R, with rho=1 chosen in the survdiff function to perform the log-rank statistical test.

Pearson correlation coefficient (PCC) was performed on the expression of *BAP1* and *MYCN* in neuroblastomas with *MYCN* amplification and *MYCN* non-amplification from gencode19 cohort of neuroblastomas–Westermann (*n* = 579) and GSE49710 (*n* = 498). Linear model was generated using the stat_smooth function from the ggplot2 R package to highlight the overall trend and association between these genes.

### Statistical analyses

All quantitative data were presented as the mean ± SD as indicated of at least three independent experiments by Student’s *t* test between group differences. *P* < 0.05 was considered as significant difference.

## Supplementary information

Supplementary BAP1-CDD

ucroped wb data-R1

aj-checklist

## Data Availability

The experimental datasets generated and/or analyzed in the study were all included in the published article.
