# Supplementary material for: Deficiency of BAP1 inhibits neuroblastoma tumorigenesis through destabilization of MYCN
Source: Cell Death Dis. 2023 Aug 5;14(8):504. doi: 10.1038/s41419-023-06030-5 (PMC10404282; doi:10.1038/s41419-023-06030-5)
Supplement: Supplementary file 1 — Supplementary BAP1-CDD [file 41419_2023_6030_MOESM1_ESM.docx]

**Supplemental Information**

**Deficiency of BAP1 inhibits neuroblastoma tumorigenesis through destabilization of MYCN**

Xiaoling Zhang, Xianling Cong, Xiangting Jin, Yu’e Liu, Tong Zhang, Xinyuan Fan, Xiyao Shi, Xiaoying Zhang, Xue Wang, Yong-Guang Yang, Xiangpeng Dai

**SUPPLEMENTAL SECTION INVENTORY**

**Figure S1 relates to manuscript Figure 1.**

**Figure S2 relates to manuscript Figure 2.**

**Figure S3 relates to manuscript Figure 3.**

**Figure S4 relates to manuscript Figure 3.**

**Supplemental Figure 1. BAP1 interacts with MYCN (related to Figure 1).**

(**A**) IB analysis of WCL derived from 293T cells transfected with the indicated plasmids.

(**B**) BAP1 and MYCN are co-localized in the nucleus of 293T cells. The cellular location of MYCN and BAP1 proteins was examined by immunofluorescence staining. DAPI was used to stain the DNA. Scale bar, 50 μm (20×) and 20 μm (40×). DAPI, 4,6-diamidino-2-phenylindole.

(**C-D**) IB analysis of whole cell lysates (WCL) and immunoprecipitates (IP) derived from 293T cells transfected with indicated constructs. Thirty hours post-transfection, cells were treated with 10 μM MG132 for 10 hours before harvesting.

**Supplemental Figure 2. BAP1 knock-down does not inhibit NB cells growth without the expression of MYCN (related to Figure 2).**

**(A)** IB analysis of WCL derived from BE2C cells infected with the indicated BAP1 shRNAs. Cells were treated with indicated concentration of MG132 for overnight before they were harvested.

**(B)** Representative images for the wound-healing assays of shBAP1 SH-EP Tet21/N cells treated with Doxycycline to deplete the MYCN. The wound edges are indicated by yellow lines. Scale bar, 100 μm.

**(C)** The quantitative results of (**B**) (n=10). The y axis represents the relative wound area. Error bars represent s.d. from ten repeats. t-test.

(**D**) Growth curves of SH-EP Tet21/N+Dox cells (Non-amplification of MYCN) with shRNA-mediated *BAP1* knockdown (shBAP1). ns, no significant difference.

**Supplemental Figure 3. Deficiency of BAP1 inhibits NB cell growth *in vivo* through MYCN (related to Figure 3).**

(**A**) Representative image for the *in vivo* tumor growth measured by detecting bioluminescence signals using imaging system at the indicated time points.

**(B)** *In vivo* tumor growth was monitored over the indicated time period by detecting and analyzing the bioluminescence signals.

**(C)** Subcutaneous tumors formed from *BAP1*-knockdown BE2C cells stably expressing MYCN were dissected.

**(D)** Representative image for the *in vivo* orthotopic tumor growth measured by detecting bioluminescence signals using imaging system at the indicated time points.

**(E)** Kidney and adrenal gland of mice bearing the orthotopic tumors from *BAP1*-knockdown BE2C cells stably expressing MYCN were dissected.

**Supplemental Figure 4. BAP1 inhibits NB cell growth (related to Figure 3).**

**(A)** Immunoblot (IB) analyses of whole-cell lysate (WCL) derived from the BE2C cells infected with indicated pBabe-BAP1. The infected cells were selected with 2-3 μg/ml puromycin for 72 hours to eliminate non-infected cells before they were harvested.

**(B)** Growth curves of BE2C cells stably expressed BAP1. **. *P*<0.01.

**(C)** Colony formation assay. Ectopic expression of BAP1 in BE2C cells displays moderate decrease in colony formation ability. The number of colonies were counted and quantified. Data was shown as mean ± s.d. for three independent experiments. *P* value was indicated in figure, *t*-test.

**(D)** Kidney and adrenal gland of mice bearing the orthotopic tumors from BE2C cells stably overexpressing BAP1 were dissected.

(**E**) The weight of kidney + tumor in Supplemental Figure 4**D**.

(**F**) The weight of kidney + adrenal gland in Supplemental Figure 4**D**.

(**G**) The body weights of the tumors bearing mice were measured at the indicated time points.

**(H)** IB) analyses of WCL derived from the SH-EP Tet21/N cells infected with indicated lentivirus BAP1 treated with or without Dox. The infected cells were selected with 2-3 μg/ml puromycin for 72 hours to eliminate non-infected cells before they were harvested.

**(I)** Growth curves of SH-EP Tet21/N stably expressed BAP1. **. *P*<0.01.

**(J)** Colony formation assay. Ectopic expression of BAP1 in SH-EP Tet21/N cells displays moderate decrease in colony formation ability. The number of colonies were counted and quantified. Data was shown as mean ± s.d. for three independent experiments. *P* value was indicated in figure, *t*-test.

**(K)** Representative images for the wound-healing assays of BAP1 overexpressed SH-EP Tet21/N cells. The wound edges are indicated by yellow lines. Scale bar, 100 μm.

**(L)** The quantitative results of **Supplemental Figure 4K** (n=10). The y axis represents the wound healing rate. Error bars represent s.d. from ten repeats. t-test.
