# Supplementary material for: Deficiency of BAP1 inhibits neuroblastoma tumorigenesis through destabilization of MYCN
Source: Cell Death Dis. 2023 Aug 5;14(8):504. doi: 10.1038/s41419-023-06030-5 (PMC10404282; doi:10.1038/s41419-023-06030-5)

Figure 1A

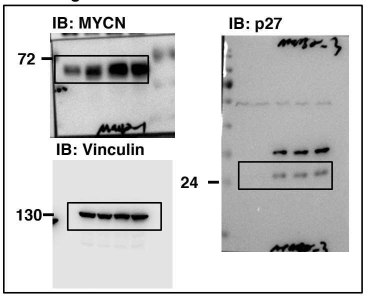

Figure 1B

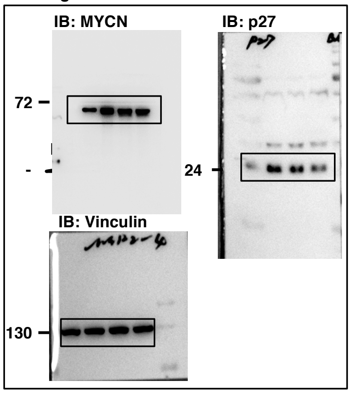

Figure 1C

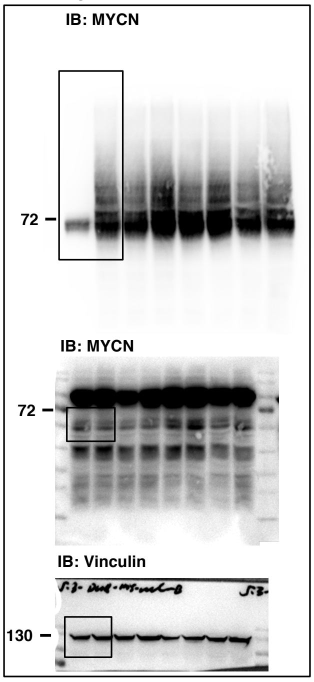

Figure 1F

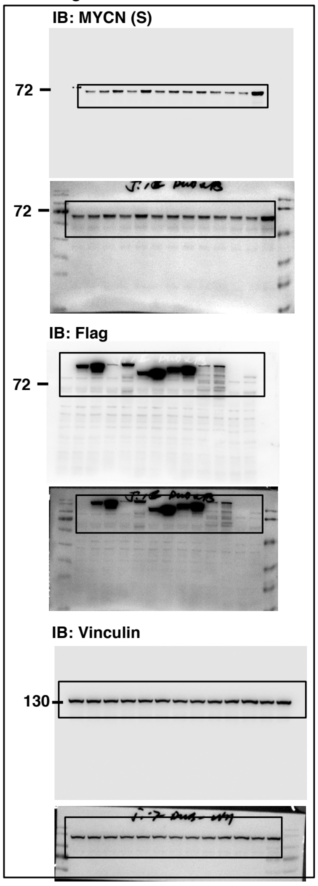

Figure 1D

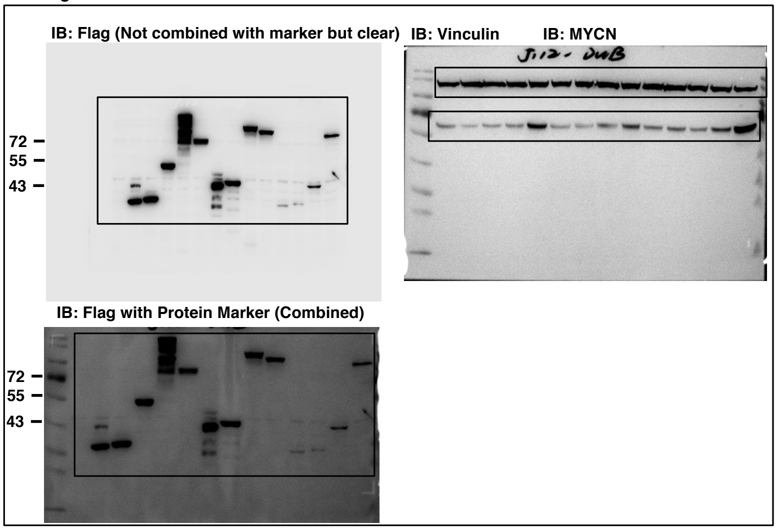

Figure 1E

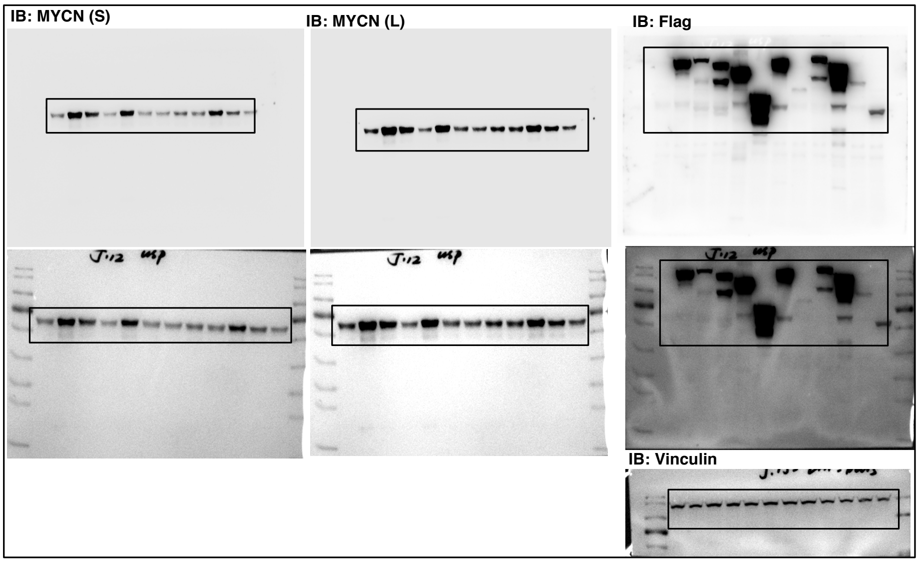

Figure 1G

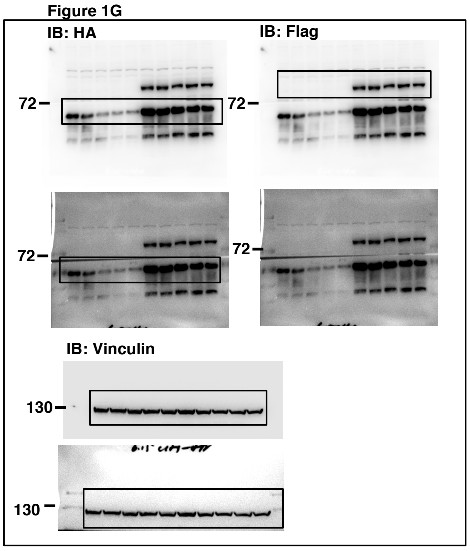

Figure 1I

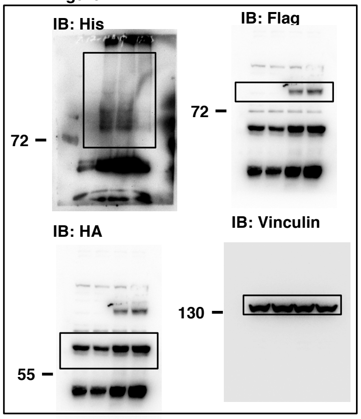

Figure 1J

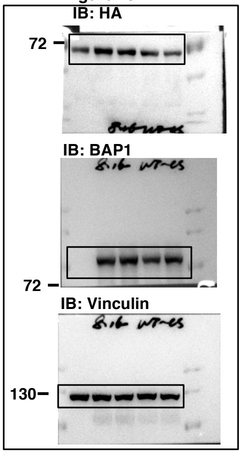

Figure 1K

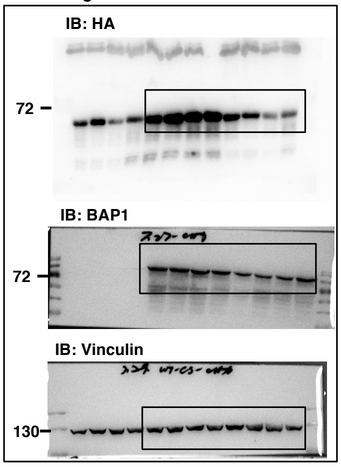

Figure 1M

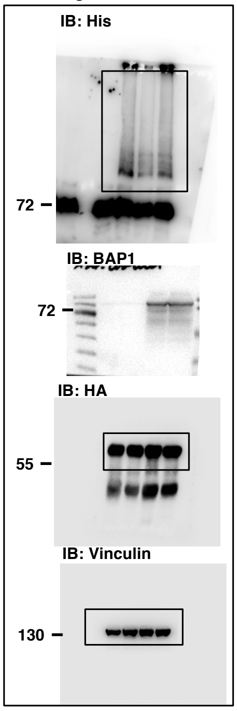

Figure 1N

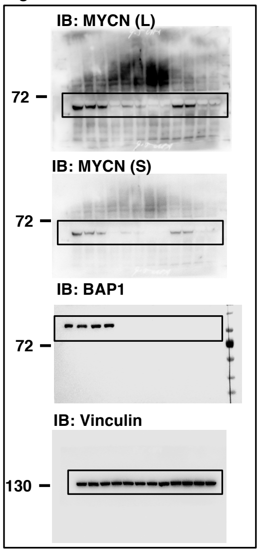

Figure 1P

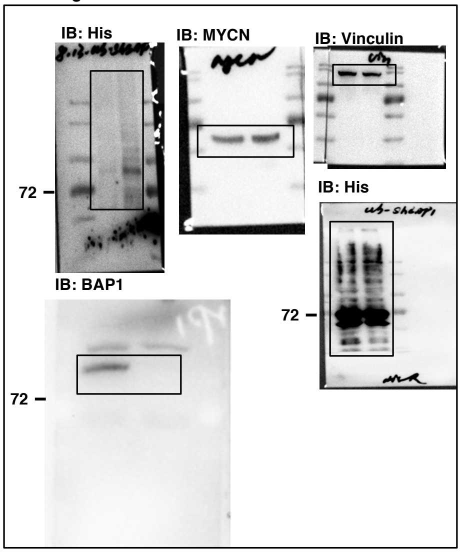

Figure 1Q

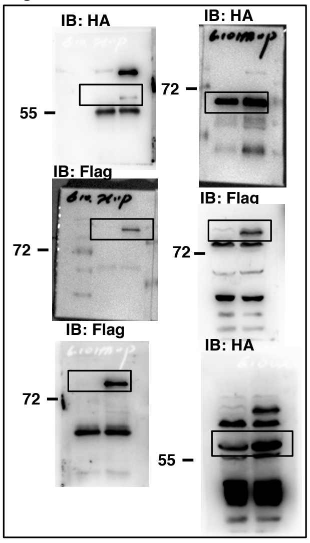

Figure 1R

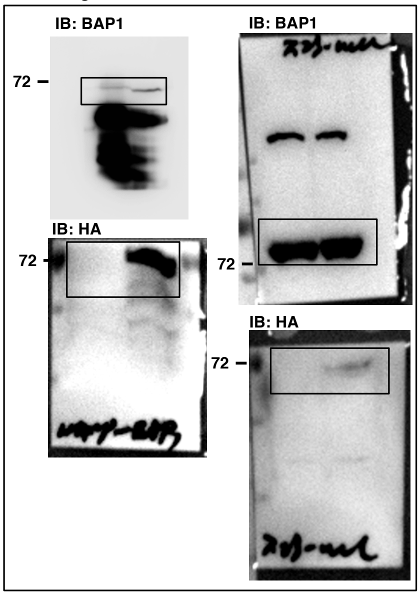

Figure 2A

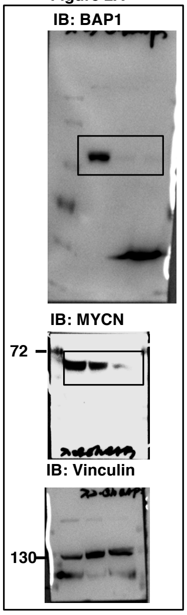

Figure 2C

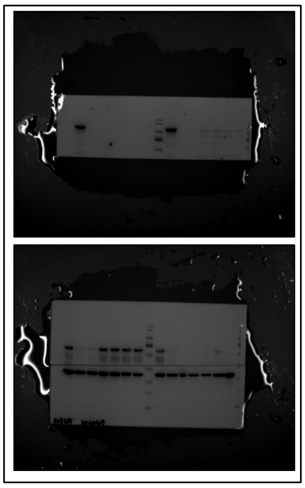

Figure 3A

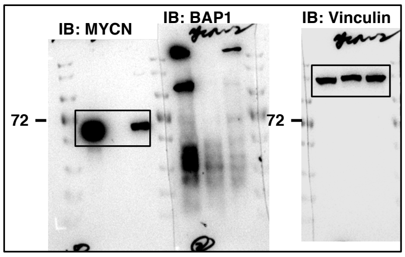

Figure S1A

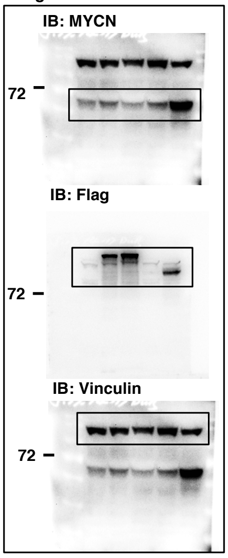

Figure S1C

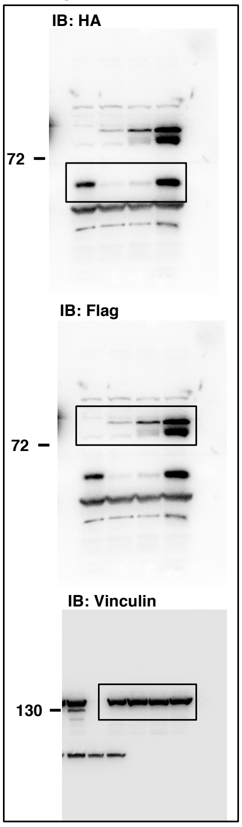

Figure S1D

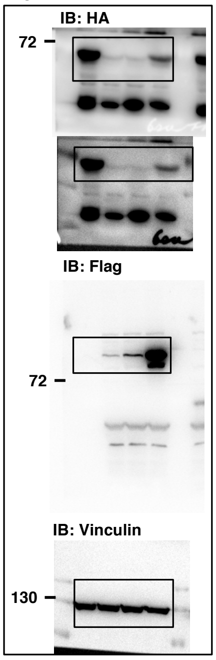

Supplemental Figure 2A

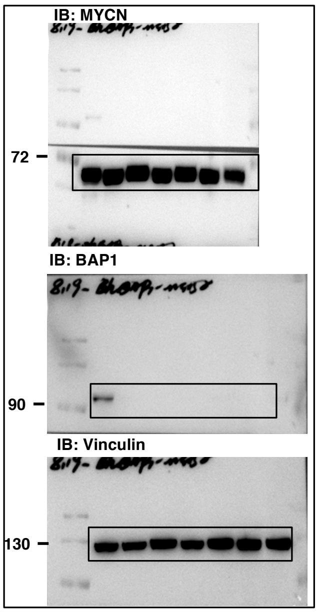

Supplemental Figure 4A

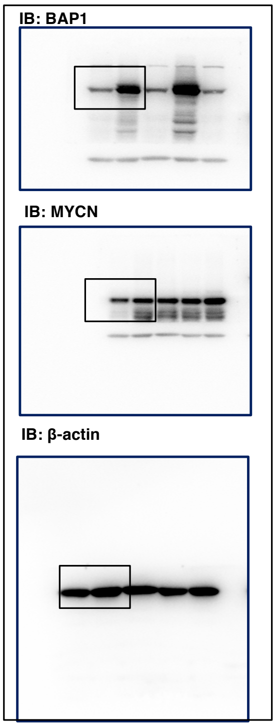

Supplemental Figure 4H

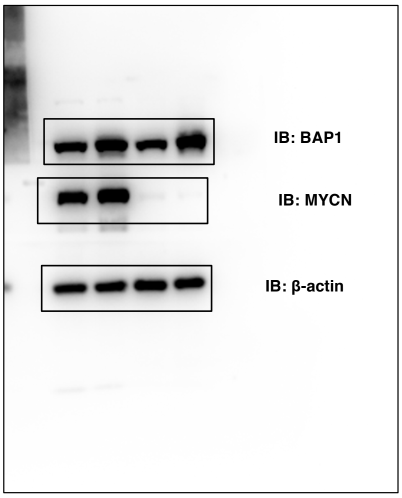

Supplement: Supplementary file 2 — ucroped wb data-R1 [file 41419_2023_6030_MOESM2_ESM.pdf]
